# Transcriptomic, Metabolomic and Ionomic Analyses Reveal Early Modulation of Leaf Mineral Content in *Brassica napus* under Mild or Severe Drought

**DOI:** 10.3390/ijms23020781

**Published:** 2022-01-11

**Authors:** Aurélien D’Oria, Lun Jing, Mustapha Arkoun, Sylvain Pluchon, Stéphanie Pateyron, Jacques Trouverie, Philippe Etienne, Sylvain Diquélou, Alain Ourry

**Affiliations:** 1 Unicaen, INRAE, UMR 950 Eva, SFR Normandie Végétal (FED4277), Normandie Université, 14000 Caen, France; aurelien.doria@unicaen.fr (A.D.); jacques.trouverie@unicaen.fr (J.T.); philippe.etienne@unicaen.fr (P.E.); sylvain.diquelou@unicaen.fr (S.D.); 2 Laboratoire de Nutrition Végétale, Agro Innovation International-TIMAC AGRO, 35400 Saint-Malo, France; mustapha.arkoun@roullier.com (M.A.); sylvain.pluchon@roullier.com (S.P.); 3 Plateformes Analytiques de Recherche, Agro Innovation International-TIMAC AGRO, 35400 Saint-Malo, France; lun.jing@roulier.com; 4 Institute of Plant Sciences Paris-Saclay (IPS2), Université Paris-Saclay, CNRS, INRAE, Univ Evry, 91405 Orsay, France; stephanie.pateyron@inrae.fr; 5 Institute of Plant Sciences Paris-Saclay (IPS2), Université de Paris, CNRS, INRAE, 91405 Orsay, France

**Keywords:** abscisic acid, genes related to transport, glutathione, ionome, jasmonic acid, mineral nutrition, proline, water deficit

## Abstract

While it is generally acknowledged that drought is one of the main abiotic factors affecting plant growth, how mineral nutrition is specifically and negatively affected by water deficit has received very little attention, other than being analyzed as a consequence of reduced growth. Therefore, *Brassica napus* plants were subjected to a gradual onset of water deficits (mild, severe, or severe extended), and leaves were analyzed at the ionomic, transcriptomic and metabolic levels. The number of Differentially Expressed Genes (DEGs) and of the most differentially accumulated metabolites increased from mild (525 DEGs, 57 metabolites) to severe (5454 DEGs, 78 metabolites) and severe extended (9346 DEGs, 95 metabolites) water deficit. Gene ontology enrichment analysis of the 11,747 DEGs identified revealed that ion transport was one of the most significant processes affected, even under mild water deficit, and this was also confirmed by the shift in ionomic composition (mostly micronutrients with a strong decrease in Mo, Fe, Zn, and Mn in leaves) that occurred well before growth reduction. The metabolomic data and most of the transcriptomic data suggested that well-known early leaf responses to drought such as phytohormone metabolism (ABA and JA), proline accumulation, and oxidative stress defense were induced later than repression of genes related to nutrient transport.

## 1. Introduction

Drought is one of the most important environmental factors that limit plant growth and productivity. In the context of global climate change, the frequency and intensity of abiotic constraints faced by plants tend to increase [1], including water deficit, which negatively impacts not only crop yield [2,3,4] but also the quality of harvested products [5,6,7,8,9]. As the world population increases exponentially, research needs to contend with a double challenge, ensuring food security and safety [10,11,12]. Hence, numerous studies have been undertaken to explore the effects of water stress across diverse plant species, and these have enabled the identification of useful molecular and metabolic traits for breeding programs targeting improvements in plant resilience to drought [13,14,15,16,17].

Water deficit induces many changes at the morphological, biochemical, physiological, and molecular levels in all plant organs [18,19,20,21]. Overall, the plant response comprises a complex and dynamic process aimed at minimizing water loss and optimizing water uptake under suboptimal water regimes. The main alterations have included perception and signaling cascades [22], gene expression regulation [23,24,25,26], phytohormone induction [27,28,29,30], reactive oxygen species scavenging [31,32,33], cell membrane structure modulation, osmolyte synthesis, and activation of ion channels [34,35], as well as metabolic modifications involving carbohydrates [36,37,38], amino acids and fatty acids [39,40]. These modifications can result in marked accelerations in phenology, promotion of the growth and architectural modification of roots, negative impacts on shoot growth, induction of leaf rolling, and changes to stomatal density and cuticular wax content [21]. Water limitation also causes cell dehydration, which disturbs cellular homeostasis and, in turn, results in ionic and osmotic stress. Further, osmotic adjustment thus implies ion movements and accumulation of carbohydrate and osmoprotectant molecules [27,39]. In order to retain water, stomatal closure is mediated by ABA accumulation in particular [28], reducing the transpiration rate but negatively affecting CO_2_ diffusion and ultimately leading to a decline in photosynthesis activity [23]. Moreover, water deficit also triggers oxidative stress, which produces reactive oxygen species (ROS) and causes damage to membranes, proteins, and DNA structures. 

Over the last few decades, the emergence of high-throughput “omics” techniques has enabled analysis of changes in the transcriptome [41,42,43,44,45], metabolome [46], and proteome [40,47,48] of tolerant and susceptible cultivars and has given access to a more holistic view of plant responses to water stress. However, to our knowledge, relatively few studies have been performed on the specific effects of drought on mineral nutrition because it is usually implicitly assumed that reduced uptake of minerals is a direct consequence of reduced growth. Indeed, the negative impact of water stress on plant mineral content is essentially explained by a reduction in the transpiration rate [34] or through the multiple functions of ions that might be missing [49]. However, recent studies attempting to decipher the mechanistic adjustments in plants growing under nutrient deficiencies [50,51,52,53] have proven the potential interest to study ion uptake and transport regulation in cases of other abiotic stresses such as drought. For that purpose, novel approaches such as examination of the functional ionome comprising all mineral elements, whether essential or non-essential for plant life [54,55], has emerged as a field of investigation [55,56,57,58,59,60,61], and recently variation in ion profiles during drought periods have been revealed [62,63,64]. For example, *Arabidopsis* is affected by moderate or severe water stress, and it shows an accumulation of Mn, Na, and Cu in leaves while Fe content is strongly reduced [62]. In recent work conducted with *B. napus* and *T. aestivum* [65], drought had an earlier and more substantial negative impact on the uptake of Fe, Zn, Mn, and Mo than for most of the other physiological and morphological parameters assessed in the two species. Moreover, it was shown that genes encoding transporters for these elements were down-regulated (in the case of Mo) or indicated a mixed pattern combining up or down-regulation (for example, Fe, Zn, and Mn). Such molecular regulation was found well before a reduction in growth, suggesting that regulation of mineral nutrition was one of the earliest responses to drought. These works pave the way for a better understanding of ionomic changes under water constraint and offer research perspectives into genes and regulatory pathways that should enable improvements in plant resilience to water stress. 

Therefore, because the mechanisms that lead to modifications of mineral nutrition in response to drought are currently poorly documented, this study in *Brassica napus* proposes to investigate them via broad transcriptomic and metabolic approaches coupled with quantification of the leaf ionome. Thus, some mechanisms already described as being involved early in adaptation to drought, such as ABA synthesis, proline accumulation, and responses to oxidative stress, were monitored kinetically alongside the expression of genes known to be involved in mineral nutrient transport. For this purpose, plants at the vegetative stage exposed to a gradual onset of water deficit managed with a high-throughput phenotyping platform were used to study the chronology of molecular and ionomic modifications in the leaves of plants facing drought conditions. 

## 2. Results

### 2.1. General Effect of Water Deficit on Growth 

**Figure 1:**
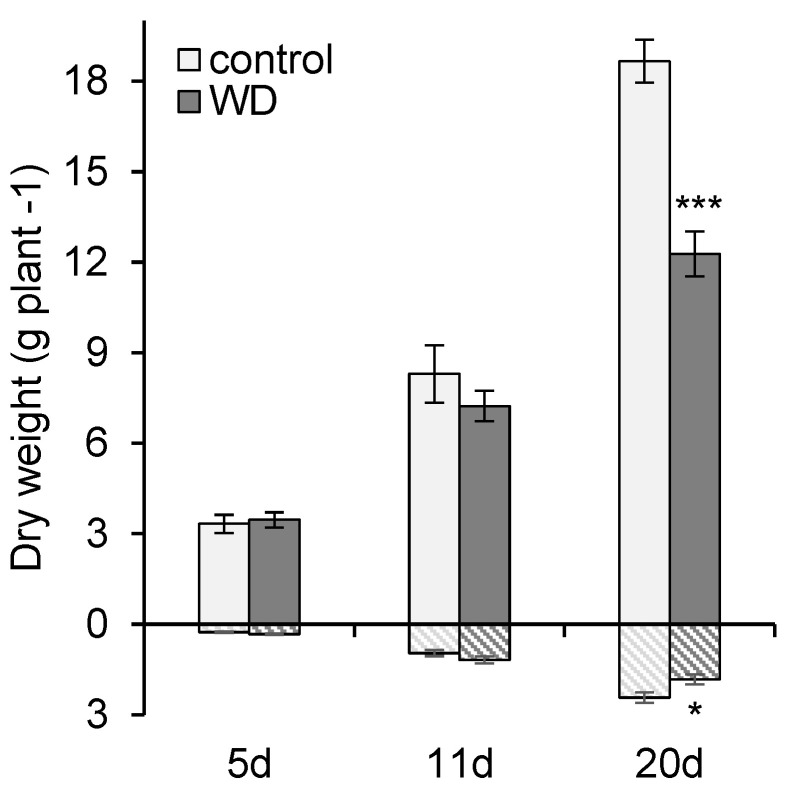
Shoot (plain) and root (hatch) biomasses of *B. napus* exposed for 5 d (mild), 11 d (severe), and 20 d (severe and extended) of water deficits (WD). Data are given as the mean ± SE (*n* = 5), and significant differences between control and plants exposed to WD are indicated as follows: *: *p* < 0.05; ***: *p* < 0.001.

**Figure 2:**
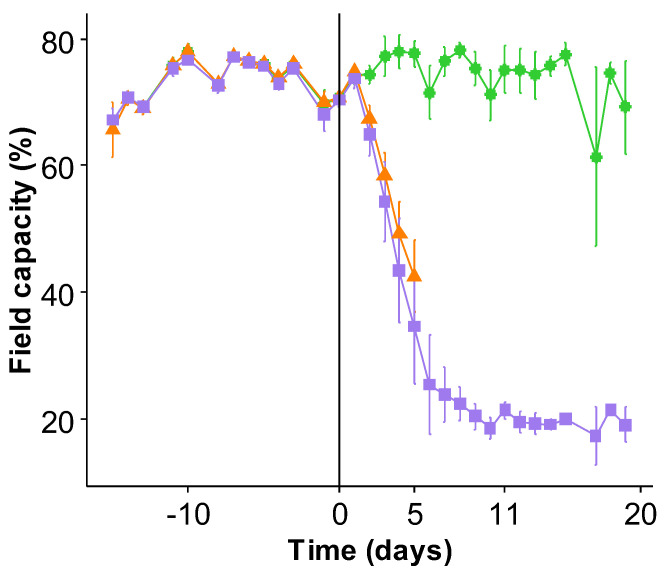
Soil water content was expressed as a percentage of field capacity (FC) during the experiment. After sowing, plants were kept at 80% FC for thirty days (until 0 d). Thereafter, well-watered control plants were kept at 80% FC until the end of the experiment, while watering was stopped at 0 d until the FC of all water deficit (WD) pots dropped to 40% FC (5 d) and then 25% FC (11 d). Consequently, from 11 d to 20 d, WD pots were held at 25% FC by automatic watering. Soil water contents, as well as the amount of water to be supplied, were automatically assessed with the high throughput phenotyping platform during the experiment, and each value corresponds to the lowest soil water content recorded daily or twice a day.

Compared to control plants, the shoot and root biomasses of *B. napus* were not significantly altered after 5 days (d) and 11 d in plants exposed to a water shortage (Figure 1), considered as either mild or severe water deficit (WD), respectively. Nevertheless, after 11 d a slight but non-significant increase in root biomass was observed compared to control plants. However, at 20 d, the WD exposure (corresponding to (i) 11 days of water shortage during which water content (WC) dropped to 25% of field capacity (FC) followed by (ii) 9 days maintained at this level by automatic watering (Figure 2), led to a significant decrease in both the shoot and the root biomass compared to the control. In shoots, this biomass decrease was mainly due to a one-third reduction in the number of newly developed leaves from the start of the WD exposure (data not shown). 

### 2.2. Overview of the Leaf Transcriptome and Metabolome of Brassica napus Exposed to Water Deficit 

**Figure 3:**
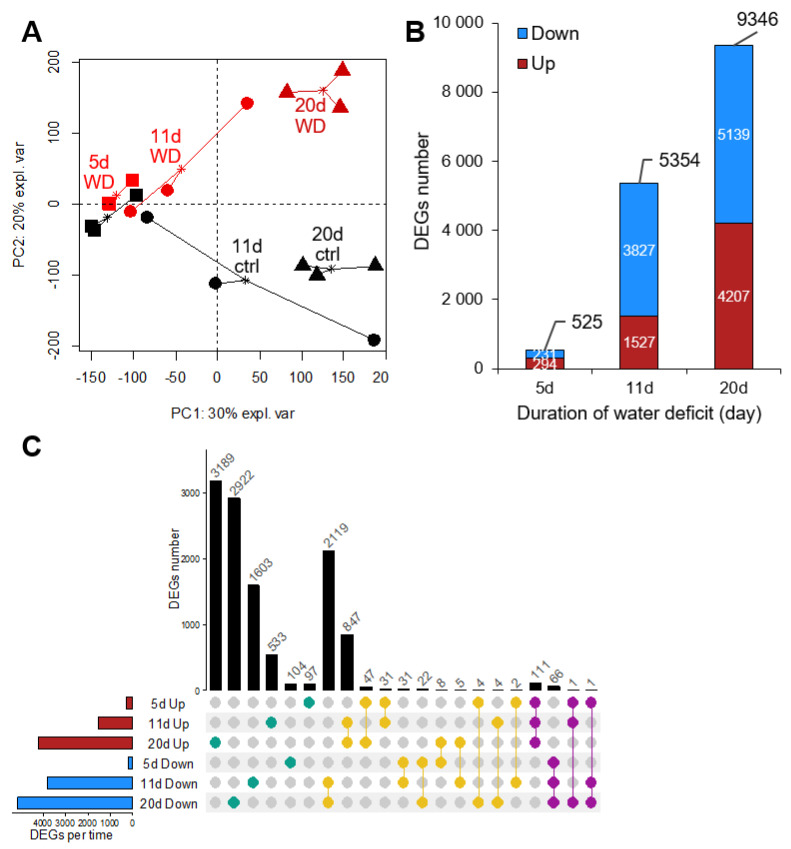
Exploratory data analysis of RNA-seq from *B. napus* leaves in response to a water deficit. (**A**) Principal Component Analysis score plot from leaf samples using normalized count data after 5 d (■), 11 d (●), and 20 d (▲) in control (“ctrl”, black) or water deficit (“WD”, red) exposed plants. (**B**) Overview of the number of total and up and down DEGs identified with RNA-seq analysis in *B. napus* exposed to 5 d (mild), 11 d (severe), and 20 d (severe and extended) water deficit. (**C**) Distribution of DEGs specific to a single date of water deficit (green) or overlapped between two (yellow) or three (purple) time points. DEGs up and down-regulated are indicated in red and blue, respectively.

Principal component analysis (PCA) of the total RNA-sequencing (RNA-seq) normalized counts showed that samples were well discriminated along with the first two components (Figure 3A). The first one (PC1), which explained 30% of the variability in leaf transcriptome, was obviously driven by a time-dependent (developmental stage) factor from 5 d to 20 d, whereas the second one (PC2), which explained 20% of the variability was driven by the water deficit applied and well separated the control from WD plants. At 5 d, control and WD samples were almost clustered, indicating a more similar transcript expression profile at this time point than at 11 d and 20 d, which was related to the growing number of differentially expressed genes (DEGs) between the different sampling points (Figure 3B,C). 

Indeed, after 5 d of water deficit, 525 genes were differentially expressed with nearly equivalent proportions of upregulated (294) and downregulated (231) DEGs (Figure 3B). In contrast, at 11 d days, the number of DEGs massively increased (10 fold), with an imbalance between upregulated and downregulated genes (2.5 fold more for the latter) in response to the WD. After 20 d, the transcriptome of WD plants compared to control was even more modulated, with 9346 DEGs (1.7 fold increase compared to 11 d) that again were in a balanced up/down ratio (1.2).

Evaluation of the distribution of the 11 747 DEGs identified across the three harvest times revealed that most of them were specifically up (3189) or down (2922) differentially expressed at 20 d (Figure 3C). Indeed, 65% of 20 d DEGs were only found at this time point, compared to 38% at 5 d and 40% at 11 d, meaning that the majority of DEGs characterized at these two first time points were also found at others. This is highlighted by the 2119 and 847 genes that were down and upregulated at both 11 d and 20 d, respectively. A much smaller number of DEGs overlapped between the three-time points and were always up (111) or down (66) differentially expressed during WD. Only a very small number of genes (22) changed their regulatory direction (i.e., up or down at different time points).

**Figure 4:**
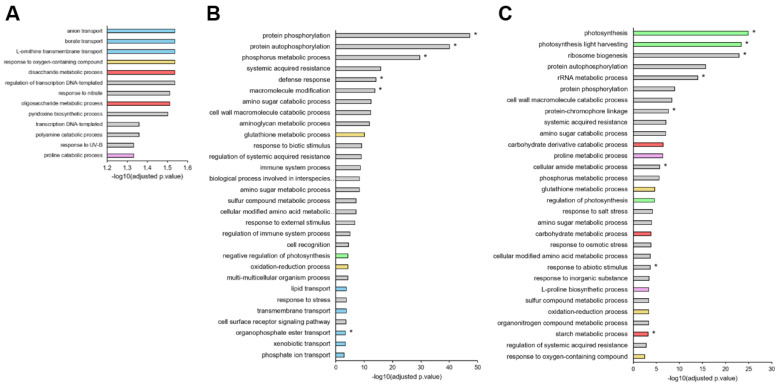
The most significantly enriched GO terms (“biological process”) from DEGs in leaves of *B. napus* exposed to 5 d (mild) (**A**), 11 d (severe) (**B**), and 20 d (severe and extended) (**C**) of water deficit. GO terms associated with transport (blue), redox homeostasis (gold), carbohydrate (red), and proline (pink) metabolism as well as photosynthesis (green) are highlighted, and terms enriched from specific DEGs at each time point are indicated with an asterisk (*).

These sets of genes were used to determine the significantly enriched gene ontology (GO) terms (in “biological process”) with the g:Profiler tool (provided in Supplementary Data S1) and then summarized by removing redundant terms with REVIGO. The most enriched terms after 5, 11, and 20 d in *B. napus* exposed to water deficit are reported in Figure 4 and are all presented in Supplementary Figures S1–S3.

Interestingly, the 525 DEGs at 5 d mostly belong to processes involved in transport (anion transport, GO: 0006820; borate transport, GO: 0046713; L-ornithine transmembrane transport, GO: 1903352), carbohydrate metabolism (disaccharide metabolic process, GO:0005984; oligosaccharide metabolic process, GO:0009311), proline (proline catabolic process, GO:0006562), and redox homeostasis (response to oxygen-containing compound, GO:1901700) (Figure 4A). Overall, these processes were also enriched at 11 d and particularly at 20 d (Figure 4B,C). At 11 d, a large proportion of specific DEGs were found to be related to phosphorylation (protein phosphorylation, GO:0006468; protein autophosphorylation, GO:0046777; phosphorus metabolic process, GO:0006793) and defense processes (defense response, GO:0006952), while the other DEGs, not specific to 11 d, were particularly related to amino acids (Figure 4B). After 20 d, in addition to previous processes cited, GO terms related to photosynthesis metabolism (photosynthesis, GO: 0015979; photosynthesis light-harvesting, GO: 0009765) seemed to be substantially enriched (Figure 4C). In fact, clear terms considered as being more specific to the water stress mainly appeared after 20 d (response to osmotic stress, GO:0006970; response to abiotic stimulus, GO:0009628; response to salt stress, GO:0009651).

**Figure 5:**
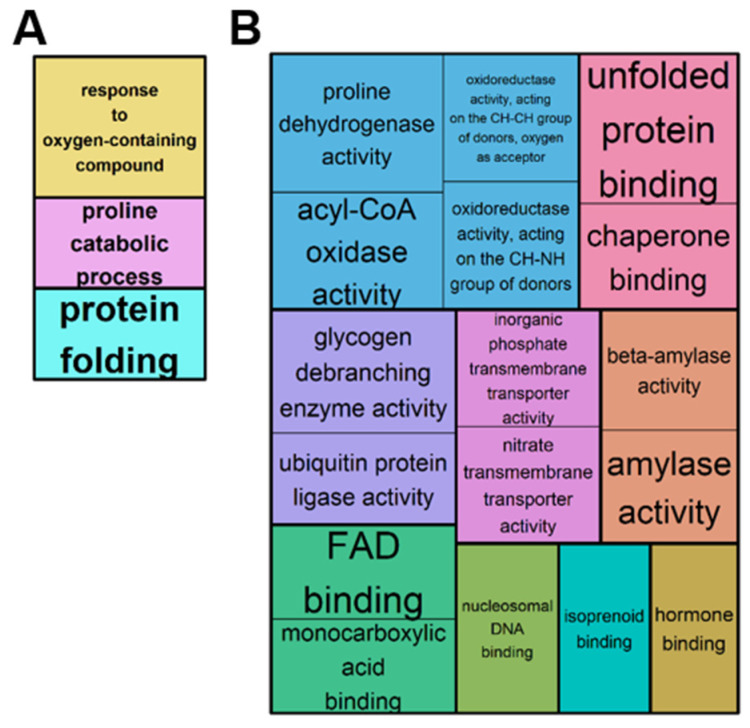
GO Enrichment analysis using g:Profiler then REVIGO for up and down DEGs overlapping between 5 d (mild), 11 d (severe), and 20 d (severe and extended) of WD compared to control plants. Significantly enriched GO terms in “biological process” (**A**) and “molecular function” (**B**) are represented in rectangles combined into superclusters with the most closely related terms, and sizes have been set to reflect the adjusted *p*-value (*p* < 0.05).

GO analysis from the 177 (111 up and 66 down) overlapping DEGs between 5 d, 11 d, and 20 d revealed that both “biological process”—and “molecular function”—enriched GO terms were related to proline metabolism, protein folding, and particularly redox homeostasis (Figure 5), in which Acyl-coenzyme A oxidase and flavine adenine dinucleotide (FAD) were involved in hydrogen peroxide synthesis (Figure 5B). Overlapping DEGs were also involved in phytohormone metabolism or steady regulation of phosphorus and nitrate transporters (Figure 5B).

**Figure 6:**
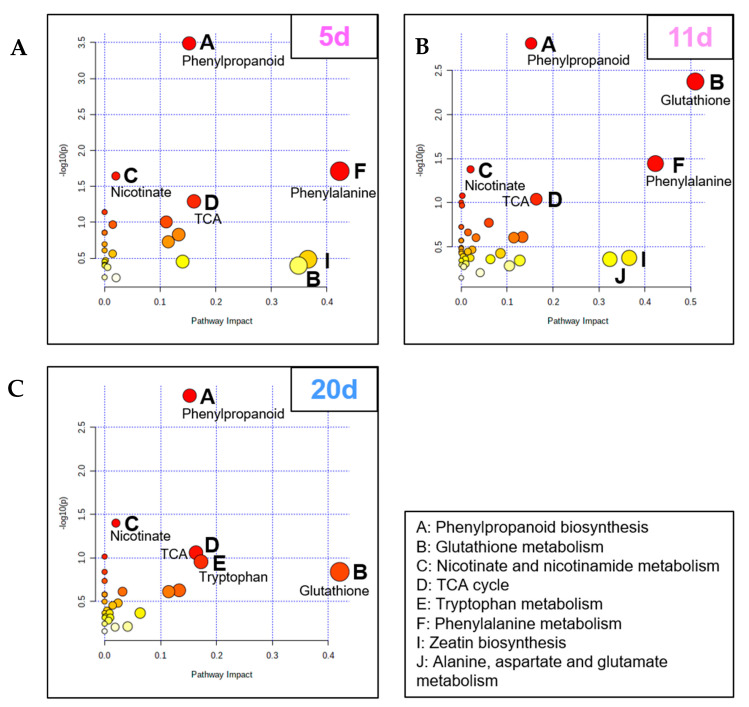
Metabolomic Pathway Analysis from leaves of *B. napus* exposed to 5 d (mild) (**A**), 11 d (severe) (**B**), and 20 d (severe and extended) (**C**) of water deficit relative to control plants. Pathways are displayed as circles whose coordinates correspond to their adjusted *p*-value expressed in −log10(*p*) (for example, *p* < 0.05 corresponds to −log10(*p*) > 1.3) and their impact. For easier reading, low to high *p*-values are displayed with a white-yellow-orange to red gradient, with white representing the lowest values and red representing the highest; the size of the circle corresponds to the pathway impact score. Only the most impacted pathways having high statistical significance scores (−log10(*p*) > 1.3) or pathway impact (>0.15) are identified.

Ultimately, an analysis of metabolite enrichment was performed using an untargeted metabolomic approach in order to determine how leaf transcriptome modulations manifested at the metabolite scale. PLS-DA (Partial Least-Squares Discriminant Analysis) performed on data from control and plants exposed to a water deficit enabled the selection of the most discriminative metabolites. They were subsequently filtered using the variable importance in projection (VIP), with VIP scores > 2. Thus, 31, 51, and 61 metabolites in positive mode and 26, 27, and 34 metabolites in the negative mode were selected at 5 d, 11 d, and 20 d, respectively. The analysis of the relevant affected pathways was performed with the Metabolic Pathway Analysis tool of MetaboAnalyst, which combines results from a powerful pathway enrichment and topology analysis. Overall, similarities with transcriptomic data were found because most of the metabolic pathways impacted corresponded to enriched GO terms. As previously observed in GO analysis, pathways involving numerous amino acids, including their derived molecules such as phenylpropanoids, were highly altered (Figure 6). For example, there were early effects on phenylalanine metabolism at 5 d and 11 d (Figure 6B,C). The glutathione pathway was altered at 11 and 20 d (Figure 6B,C), while tryptophan was only at 20 d (Figure 6C). Nicotinate and nicotinamide metabolism, as well as TCA cycle metabolite enrichments, were constantly found during the water stress applied. 

### 2.3. Chronology of Metabolic Events: The Case of ABA, JA, Glutathione, and Proline

Because both the transcriptomic (Figure 4) and metabolomic (Figure 6) data revealed early plant responses to water shortage, and especially through processes involving phytohormones, glutathione, and proline metabolisms, fine kinetic monitoring of sets of genes and metabolites related to the relevant pathways was performed.

**Figure 7:**
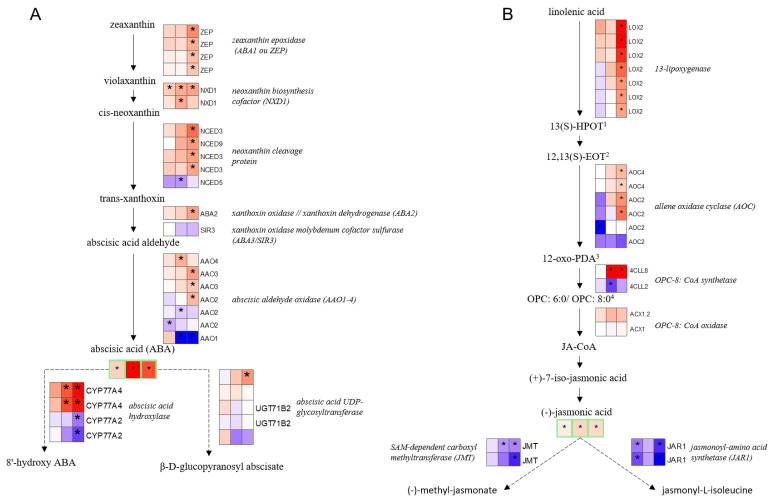

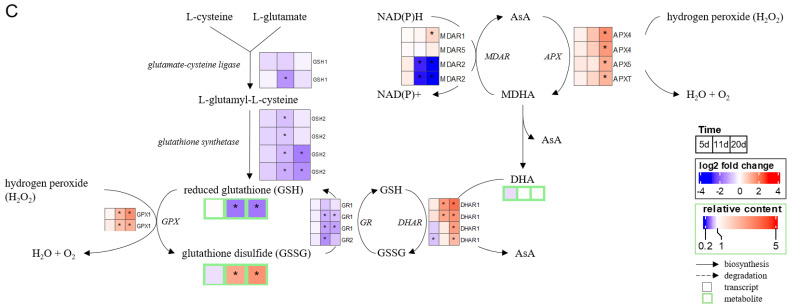
Summary schemes showing the main changes in leaves of *B. napus* induced by water deficit at the transcriptomic and metabolomics levels relative to control plants. *B. napus* plants were exposed to water deficit for 5 d (mild), 11 d (severe), and 20 d (severe and extended). Color-filled boxes indicate the log2 fold change of DEGs or the relative content of ABA (**A**), JA (**B**), and reduced and oxidized glutathione (**C**). Up and down modulations are indicated in red and blue, respectively. Significant variations in gene expression or metabolite levels between control and plants exposed to WD are indicated with an asterisk (*) for an adjusted *p*-value < 0.05. Solid and dashed lines indicate biosynthesis and degradation pathways, respectively. ^1^ hydroperoxylinolenic acid; ^2^ 12,13(S)-epoxylinolenic acid; ^3^ 2-oxo-phytodienoic acid; ^4^ oxo-pentenyll-cyclopentane; APX: ascorbate peroxidase; AsA: L-ascorbate; DHA: dehydroascorbic acid; DHAR: dehydroascorbate reductase; GPX: glutathione peroxidase; GR: glutathione reductase; MDAR: monodehydroascorbate reductase; MDHA: monodehydroascorbate.

At first, an early accumulation of abscisic acid (ABA) and jasmonic acid (JA) was observed as early as 5 d in leaves of *B. napus* exposed to water deficit (Figure 7A,B). However, at this time point, almost no variation in the transcript level of genes involved in either the biosynthesis or degradation pathways of the two hormones was noted. For JA, this accumulation may be linked to the down regulation of genes involved in hormone degradation due to conjugation with L-isoleucine (Figure 7B). Overall, the accumulation of ABA at 11 d was greater than at 5 d, but again was correlated with very few significant DEGs, even when the neoxanthin biosynthesis cofactor was clearly overexpressed. JA showed a similar trend at 11 d. Finally, at 20 d only, a myriad of genes involved in the biosynthesis of both phytohormones were overexpressed, while the degradation pathways of JA were repressed. For ABA, the expression levels of genes involved in degradation pathways did not allow the identification of a clear trend.

The levels of reduced and oxidized glutathione were not altered in plants exposed to a 5 d water deficit (Figure 7C), while the expression level of genes involved in its biosynthesis remains unchanged under WD and this, was also the case for genes involved in the hydrogen peroxide removal process. As revealed in the metabolomic overview, the level of reduced glutathione was decreased (two-fold), and the level of oxidized glutathione was significantly increased (three-fold) from 11 d up to 20 d. Interestingly, at the same time, the expression of genes involved in glutathione biosynthesis was mainly downregulated, whereas genes coding enzymes involved in the removal of hydrogen peroxides such as ascorbate peroxidase and glutathione peroxidase were overexpressed (Figure 7C). 

**Figure 8:**
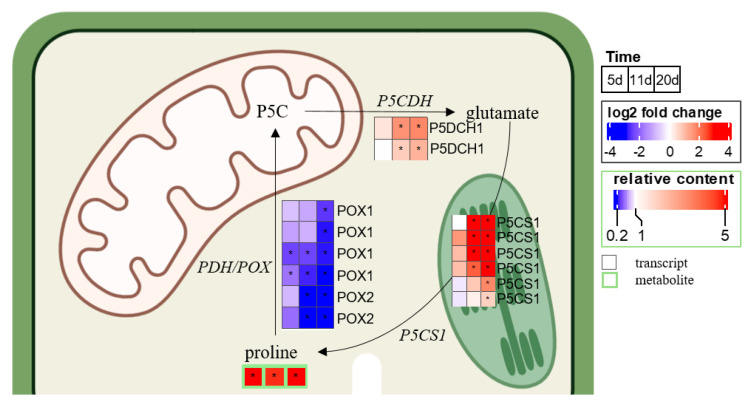
Log2-fold changes in DEGs and the proline content in leaves of *B. napus* exposed to water deficit for 5 d (mild), 11 d (severe), and 20 d (severe extended), relative to control plants. Significant variations between control and plants exposed to WD are indicated with an asterisk (*) for adjusted *p*-value < 0.05. P5C: pyrroline-5-carboxylate; P5CDH: delta-pyrroline-5-carboxylate dehydrogenase; P5CS1: pyrroline-5-carboxylate synthase; PDH: proline dehydrogenase; POX: proline oxidase.

Finally, relative to control plants, the level of proline assayed in the leaf samples increased at 5 d, then skyrocketed at 11 d and remained considerably elevated at 20 d, by 5, 150, and 30 fold, respectively, in plants exposed to water deficit (Figure 8). This was concomitant with the downregulation of genes involved in proline catabolism to pyrroline-5-carboxylate (P5C) via the sequential action of proline dehydrogenase (PDH) or proline oxidase (POX). By contrast, genes coding a delta-pyrroline-5-carboxylate dehydrogenase (P5CDH), which converts P5C to glutamate, and a chloroplastic pyrroline-5-carboxylate synthase (P5CS1) involved in proline biosynthesis were significantly overexpressed. 

### 2.4. Early Effects of Water Deficit on the Leaf Ionome

**Figure 9:**
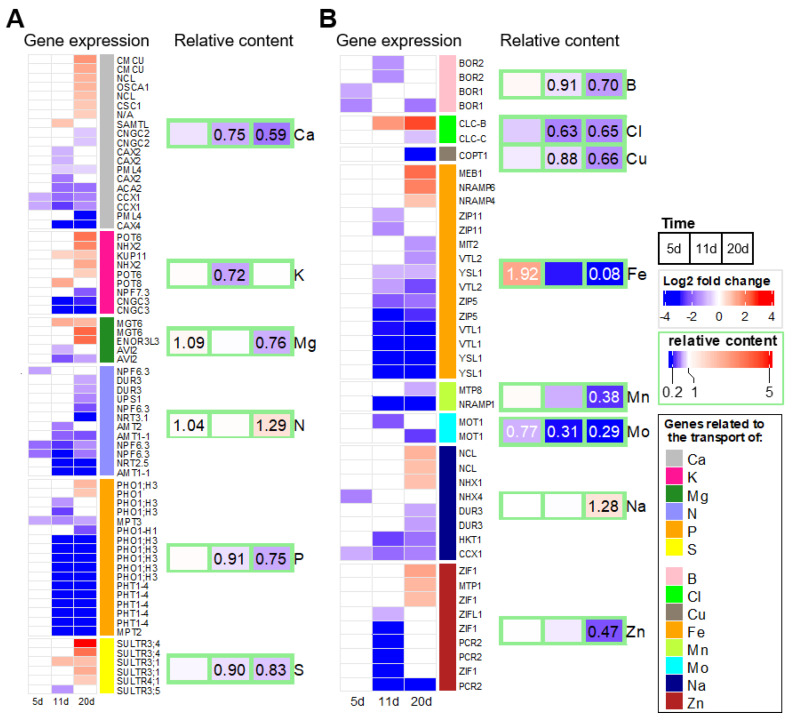
Expression profiles of 114 genes encoding macronutrient (**A**) or micronutrient (**B**) transporters and changes in mineral nutrient contents in leaves of *B. napus* exposed to 5, 11, and 20 d of water deficit, relative to control plants. Log2 fold changes in DEGs are colored in red (up) or blue (down), and non-significant variations in gene expression are blank (adjusted *p*-value < 0.05). Boxes indicate the relative content of nutrients as the ratio WD/control, and only significant variations are reported.

GO enrichment analysis also revealed an early response involving anion transporters in leaves of *B. napus* exposed to water deficit (Figure 4A). In order to deepen the understanding of this response, DEGs coding ion transporters and recently reported in a review [66] as genes with a known direct relationship to the plant elemental content were selected. This list was supplemented with a set of DEGs extracted from the “ion transport” (GO:0006811) GO term in order to study the expression profiles of 114 genes listed in Supplementary Data S2. The expression profiles of these genes confirmed that, as early as 5 d, the expression levels of nine genes associated with Ca, N, P, B, and Na transport were down-regulated (Figure 9). As previously described (Figure 5), genes associated with N and P were still substantially down-regulated for the duration of the water shortage (Figure 9A). However, the down regulation of these nine genes had no major impact on the element content examined in leaves at 5 d, whereas Fe and Mo content were already significantly increased and decreased, respectively (Figure 9B). At 11 d, the expression of a larger number of genes (64) was altered, the majority being downregulated, while significant decreases in macronutrient (Ca, K, P, and S) and micronutrient (B, Cl, Cu, and Mo) contents were observed. Subsequently, the expression of 89 genes at 20 d was significantly modulated with a simultaneous up and down regulation of genes associated with the transport of Ca, K, Mg, S, Cl, Fe, Na, and Zn. These gene expression modulations were concomitant with a large decrease in the contents of almost all nutrients in *B. napus* leaves, except N and Na. At 20 d, the largest decreases in leaf nutrient content were found for Fe (−92%), Mo (−71%), Mn (−62%), and Zn (−53%), while the only mineral nutrients showing higher leaf concentrations were N and Na (+29%), despite a significant down expression of genes involved, for example, in N uptake, regardless of the N form involved (nitrate, ammonium or urea, as seen in Figure 9A and Supplementary Data S2). 

## 3. Discussion

In the context of climate change associated with world population growth, the double challenge of maintaining or improving yield as well as nutritional quality [6], including the mineral content of the harvested products, requires a greater understanding of plant responses to biotic stresses. This is particularly true for Brassica napus, a cultivated species with a high requirement in terms of fertilization [67,68] and whose seed quality may be impaired by deficiencies [69,70,71], heat stress [72,73], elevated CO_2_ [74], or drought [75]. While the effects of water stress on morphological, physiological, metabolomics, and molecular traits have been widely documented [14,18,19,20], little attention has been focused on its effects on mineral nutrition. A previous study [65] has shown in Brassica napus and Triticum aestivum that net uptake of Fe, Mn, Zn, and Mo was strongly reduced by water deficit and resulted in a drop in the contents of these nutrients in leaf tissue. Consequently, the leaf ionome has been used as a proxy to evaluate mineral nutrition. The main purpose of this study was then to position the alteration in the leaf ionome due to water deficit within processes that are already broadly documented. Therefore, the concomitant analyzes of transcriptomic, metabolomic, and ionomic data at the leaf level and after a gradual onset of water deficit were undertaken before a significant decrease in plant biomass (Figure 1). 

### 3.1. Leaf Mineral Content Is Affected Early by Drought 

In this study, the effect of water deficit on mineral nutrition was examined in two stages: the first stage consisting of withholding water until the soil water content dropped to 40% and then 25% of FC, reached after 5 d and 11 d respectively, and then a second stage with FC kept at 25% from 11 d to 20 d (Figure 2). Consequently, the three harvests can be considered as (i) mild, (ii) severe, and (iii) severe and extended water stress, respectively. These water regimes only led to a significant reduction in plant biomass at the last time point studied, i.e., under severe and extended water stress (20 d) (Figure 1). Interestingly, after only 5 d of water shortage, the mineral content of the leaves was found to be already altered for two elements, Fe whose content doubled, and Mo, whose content dropped by a third compared to the well-watered control plants (Figure 8B). After 11 days, the whole mineral composition of the leaves was negatively impacted overall by the water shortage before any effect on plants biomass (Figure 8). Lastly, at 20 d, plants exposed to an extended water deficit showed a significant biomass reduction, whereas, except for N and Na, the leaf ionome composition showed significant decreases in almost all element contents, particularly Fe, Mo, Zn, and Mn. These results are in line with a previous meta-analysis [76], showing that during drought imposed as a “prolonged drying”, the N content was less altered than P and led to an increase in the N/P ratio, as also observed in the C3 grasses *A. pratensis* and *H. lanatus* [46]. On the other hand, a large increase in K concentrations has been shown in plants subjected to water stress [77], and initially, this was not observed in this study within leaves (Figure 8A). However, in the current work, the K content was close to the levels present in control plants, and this was probably linked to the fact that several genes encoding K transporters were overexpressed from 11 d (Figure 8A). Moreover, this could further explain the increase in leaf Na content (Figure 9B), a phenomenon also reported in the literature [62,78], which may result from the replacement of K by Na in photosynthesis and carbohydrate metabolic pathways [79] and as a result of K transporters converting into high-affinity Na transporters [80]. Unfortunately, very few studies have investigated the effects of water stress and assessed the uptake or tissue content of other elements, particularly micronutrients, which is necessary to progress towards ionomic studies. For example, a gradual decrease in Ca, Mg, and Fe have been reported in *Arabidopsis* [62] exposed to a mild (50% FC) or a severe (25% FC) drought, and this was also observed in the present study with rapeseed. Similarly, the same authors reported an early drop in the Na concentration followed by an elevation with increasing drought severity, the latter observation being found here for extended and severe water deficit. By contrast, an increase in Mn and Cu leaf contents reported [62] was not found under these experimental conditions. Paralleling other ionomic studies [63,64], this work supports an overall negative effect of water stress on the contents of almost all elements, and this was not obviously the result of a decrease in plant growth. Furthermore, these studies all agreed on the fact that the increase in intensity and/or duration of water stress had an overall negative impact on the ionomic content in plant tissue, including the edible parts, as also reported in the context of other abiotic stresses such as elevated CO_2_ [74]. Nevertheless, these studies have also revealed opposite trends in the way water stress altered the content of some elements [81], particularly under mild or short drought [62], and this underlines the influence of the experimental conditions on ionomic changes as previously reported [76] and demonstrated the need for homogenization of methodologies so that comparable results can be obtained. 

### 3.2. At the Molecular Level, a Specific Down-Regulation of Genes Encoding Transporters Precedes the Modification of Leaf Ionomic Content

Overall, the result of the processes that took place between 5 d and 11 d at leaf level demonstrates a significant decrease in the content of a large number of elements together with the negative regulation of the expression of genes encoding the associated transporters. Indeed, only 5 d after the start of withholding water, a very early downregulation of the expression of several genes encoding N (NPF 6.3), Ca (CCX1), P (MPT3), B (BOR1), and Na (NHX4 and CCX1) transporters was observed, therefore preceding the modification of the associated contents in leaves that occurred after 11 d or 20 d (Figure 9). Six days later (11 d), these genes remained steadily downregulated alongside a significant decrease in Ca, P, and B content, while the N and Na contents remained similar to control plants. By contrast, at 5 d, the water deficit applied disturbed the Fe and Mo content, but the genes encoding the associated transporters were not impaired. In fact, it has been shown in *Chlamydomonas reinhardtii* that the transcription of the Mo transporter CrMOT1, also identified in *A. thaliana* [82], does not depend on the availability of Mo but is regulated by N levels [83], which seemed to have undergone little alteration here. Surprisingly, the short-term increase in Fe content in leaves was not associated with an overexpression of genes encoding Fe transporters in order to promote cell entry or vacuolar unloading. Conversely, Fe and Mo contents were negatively impaired from 11 d onwards, i.e., under severe water deficit, and genes encoding their associated transporters were considerably downregulated, as reported for Fe in a previous *A. thaliana* transcriptomic analysis [42].

Although it could be hypothesized that the lower elemental contents observed were perceived by the plant as a deficiency, the response at the transcriptomic level did not seem to match this because nutrient deficiencies are usually characterized by overexpression of a myriad of ion transporters. Indeed, the overexpression of IRT1, considered the main transport pathway of Fe in the plant, is a well-known feature of Fe deficiency [84], as is the overexpression of other members of the ZIP multigene family such as IRT3, ZIP2, and ZIP4 in Zn or Cu deficiency [84]. This is also the case for *NRAMP1*, an essential Mn transporter overexpressed during Mn deficiency in *A. thaliana* [85]. The overexpression of all these genes, which is known as a hallmark of nutrient deficiency, was not observed here when plants faced a water deficit, despite a massive decrease in the concentration of these elements in the leaves. Furthermore, the ionome seems to be finely regulated during a deficiency [56]; for example, in leaves of *A. thaliana*, Fe content remains constant, whereas under the water deficit tested conditions, the Fe content eventually declined, which was probably a consequence of a broad downregulation of the genes encoding Fe transporters (Figure 9B). Results suggest that during water stress, the response of plants in terms of expression of genes encoding nutrient transporters is specific and differs from a nutrient deficiency, even though drought results in a significant decrease in the overall content of elements. This hypothesis is also supported by a previous work [42], where a transcriptomic analysis of the differential response of *A. thaliana* roots and shoots also revealed that genes involved in Fe uptake machinery, such as *IRT1*, *IRT3*, and ferric reduction oxidase 2 (*FRO2*), were downregulated at the root level after five to nine days of drought. These authors reported a downregulation of the transcription factor *FIT1*, which specifically controls the expression of *FRO2* and *IRT1* in roots, and suggested that this regulation under drought alters the distribution of Fe within the plant. Although the expression of genes involved in Fe uptake may be subject to the control of phytohormones such as ABA and JA [86], these results evidenced an early and concomitant accumulation of ABA, JA and Fe in leaves of *B. napus* without variation in Fe transport-associated gene expression. Since it has been suggested [87] that ABA increases root to shoot translocation of Fe during deficiency in *Arabidospis*, this might explain the transient and early Fe accumulation observed here under drought, but in that case, the mechanism still remains to be investigated. 

### 3.3. Global Transcriptomic Analysis Revealed That Downregulation of Mineral Nutrition Is Elicited before Phytohormone and Proline Metabolism or the Occurrence of Oxidative Stress

It was firstly found using PCA analysis (Figure 3A) that transcriptomic data managed to discriminate plants exposed to water deficit compared to control plants. This was due to a large modification of transcriptome profiles resulting from mild, severe, and severe extended water deficits with 525 (231 down, 294 up), 5454 (3827 down, 1527 up), and 9346 (5139 down and 4207 up) DEGs identified, respectively (Figure 3B). Numerous transcriptomic and metabolomic analyses have tried to decipher the response of *Arabidopsis thaliana* [42], wheat (*Triticum astivum*) [36,40,88], potato (*Solanum tuberosum*) [89,90], common bean (*Phaseolus vulgaris*) [91], cotton (*Gossypium herbaceum*) [92,93], tomato (*Solanum lycopersicum* L.) [94], or rapeseed (*Brassica napus* L.) [41,45] under drought. GO analysis in the majority of these studies reported large changes in the following metabolic classes: phytohormone regulation, defense responses, osmotic stress, salt stress, abiotic stimulus, oxygen-containing compounds and oxidative stress, and the metabolism of carbohydrates, amino acids, and lipids. Interestingly, a substantial number of these studies also reported GO terms related to ion transporters [41,42,88,89,91,93], but to the best of our knowledge, this aspect has not been an in-depth focus, as it was done for rapeseed subjected to saline stress [44]. In this study, it was thus decided to explore the effect of a water deficit on mineral nutrition in greater depth. Special attention was paid to the shift in the leaf ionome and the expression of genes coding ion transporters so as to compare their earliness with ABA, JA, glutathione, and proline responses, which comprise the most frequently reported processes in the studies cited above. Indeed, the GO analysis revealed that among the most enriched terms, ion transporters and especially borate transport are among the earliest forms of altered metabolism in plants subjected to drought (Figure 4), and this was confirmed through assessment of the gene expression of ion transporters (Figure 9). However, checking the increased expression of these transporters by proteomic analysis could be difficult for two main reasons: Firstly, these transport proteins may be accumulated at a very low level; secondly, the correlation between protein synthesis and corresponding gene expressions have shown low correlations, as has been reported for the wheat [95]. The harvest of plants after 5, 11, and 20 d revealed an alteration in the oxygen-containing compound content as early as 5 d (Figure 4). Indeed, under drought, it has been reported that oxidative stress is accompanied by the formation of reactive oxygen species (ROS) such as superoxide, hydrogen peroxide, and hydroxyl radicals, which cause cellular damage and inhibition of photosynthesis [20]. At the metabolomic level, glutathione metabolism was progressively altered, but mainly after 11 d and 20 d of WD (Figure 6B,C), while the nicotinamide metabolism, which is the precursor of NAD (nicotinamide adenine dinucleotide) and NADP (nicotinamide adenine dinucleotide phosphate), two cofactors involved in all of the plant’s redox reactions, was always altered irrespective of the severity or the length of the water deficit (Figure 6). Nevertheless, at the molecular level, there was no evidence before 11 d that a variation in gene expression was involved in glutathione synthesis, nor was there evidence of enzymes that scavenge hydrogen peroxide (Figure 7C). 

Since ROS damages membranes and drought decreases the leaf water content, the response of plants comprises osmotic adjustment, and especially via osmoprotectants including carbohydrates and proline biosynthesis. In accordance with a reported meta-analysis [39], raffinose was significantly accumulated in leaves in this study (10 fold, data not shown) while proline greatly increased (150 fold) at the same time (Figure 8). Indeed, proline accumulation has been reported to play a crucial role in plant stress tolerance and may contribute to drought tolerance [96,97]. This accumulation peak was concomitant with the downregulation of genes contributing to proline degradation to pyrroline-5-carboxylate (P5C); and overexpression of genes orthologous to *A. thaliana* P5CS1, which encodes the delta1-pyrroline-5-carboxylate synthase enzyme that catalyzes the rate-limiting step of proline biosynthesis [98]. In *B. napus*, *P5CS1* was notably identified among putative candidate genes for water stress tolerance [43]. If proline was accumulated early (at 5 d), at the molecular level again, almost no gene expression variation was observed. 

This data supports the notion that regulation of proline and accumulation of carbohydrates may be mediated by ABA accumulation [32], and some authors [27] have proposed a model of crosstalk between ABA and carbohydrate metabolism being involved in hexose accumulation, mainly due to starch degradation. Surprisingly, the GO analysis undertaken here revealed no terms linked to phytohormones even though early ABA and JA accumulations were found. Moreover, their early accumulation might not have resulted from the overexpression of genes involved in their biosynthesis or down-regulation of genes involved in the degradation of ABA (Figure 7A,B). Instead, increased ABA concentrations in leaves could have resulted from cell dehydration rather than its de novo synthesis [99]. 

Overall, the results of this study clearly show that during drought, mineral nutrition is affected early at the leaf level in regards to ionomic content and downregulation of ion transporters. While often attributed to reductions in K and Ca contents, which are the two major elements involved in osmotic adjustments and the signaling cascade [44], respectively, in most cases, the contents of all elements seemed to become impaired eventually, but without the transcriptomic responses that are normally found under nutrient deficiencies [53]. This underscores the need for further studies to decipher the mechanisms involved in such differential regulation. Among the future targets is the role of phytohormones such as ABA in regulating plant nutrient uptake, which was previously revealed through exogenous application studies [100], or examination of the differential expression of upstream transcription factors [41]. Additionally, since numerous studies have highlighted the differential regulation that occurs in shoots and roots during drought [41,42], new research addressing all plant compartments may allow explanation of such leaf ionomic variations. 

## 4. Materials and Methods

### 4.1. Plant Material and Growth Conditions

Seeds of rapeseed (*Brassica napus* cv. Trezzor) were germinated in trays filled with a potting soil mixture (NFU 44551, type 992016F1, Falienor S.A., Vivy, France) composed of sandy loam (40% *v*/*v*) and peat moss (60% *v*/*v*) supplemented with clay (40 kg m^−3^) and NPK fertilizer (0.7 kg m^−3^ PG-MIX 14-16-18) with a soil solution at pH 5.9 (+/−0.2) (composition given in Supplementary Table S1) in a growth chamber (16 h/8 h light/dark cycle at 20 °C and 18 °C respectively, at 80% relative humidity). After the emergence of the second leaf, each seedling was transplanted into a 6.5 L pot (20.6 cm diameter) filled with 5000 g of the potting soil cited above and watered to 80% of field capacity (FC), and was then placed in greenhouse conditions (16 h day/8 h night at 25 °C/20 °C) in the high-throughput plant phenotyping platform of the Centre Mondial de l’Innovation (Saint-Malo, France), with natural light supplemented with high-pressure sodium lamps (MST SON-T PIA Plus 400 W, Philips, Amsterdam, The Netherlands) to ensure at least 250 µmol m^−2^ s^−1^ of PAR at canopy height. 

Based on the data provided by automatic weighing twice a day, pots were then watered to maintain 80% FC and fertilized twice with 100 mL of a modified Hoagland solution (corresponding to 40 kg N ha^−1^, composition available in Supplementary Table S2) to ensure the plants’ needs until the end of the experiment, according to estimations from previous experiments [61,101,102].

Thirty days after sowing, well-watered control plants were kept at 80% FC, while watering was stopped until FC dropped to 40% FC (after 5 d) and then 25% FC (after 11 d) (Figure 2). Afterward, from 11 d to 20 d, water inputs were restarted in order to maintain 25% FC until the end of the experiment. 

Plants were sampled at 5 d, 11 d, and 20 d with five replicates, each consisting of two plants. At 0 d, the last-developed aboveground tissues were identified using a marker pen on the petiole so as to only examine leaves that were developed during water shortage for analysis. The number of newly developed leaves from 0 d and the total leaf number per plant were recorded. Each fresh sample was weighed and separated into two homogeneous batches: one immediately frozen in liquid nitrogen before being stored at −80 °C for transcriptomic and metabolomic analyses, the other oven-dried for 72 h at 65 °C for dry weight determination and element analysis. 

### 4.2. RNA Extraction, Reverse Transcription, and Q-PCR Analyses

According to the previously described protocols [103], total RNAs were extracted from 200 mg of fresh leaves developed during a water shortage and powdered prior to analysis using a mortar containing liquid nitrogen. In brief: 750 μL of hot phenol (80 °C, pH 4.3) and 750 μL of extraction buffer (0.1 M TRIS, 0.1 M LiCl, 0.01 M EDTA, 1% SDS (*w*/*v*), pH 8) were added and the mixture was vortexed for 40 s. Then 750 μL of chloroform:isoamylalcohol (24/1: *v*/*v*) was added before centrifugation at 15,000× *g* for 5 min at 4 °C. The supernatant was recovered, and 750 µL of a 4 M LiCl solution (*w*/*v*) was added for nucleic acid precipitation overnight at 4 °C. The mixture was then centrifuged at 15,000× *g* for 20 min at 4 °C, the supernatant was removed, and 100 μL of sterile water was used to suspend the pellet. Extracted RNAs were purified by DNAse digestion using RNA Clean and Concentrator kits (Zymo Research, Irvine, CA, USA). Total RNA quantification was evaluated by spectrophotometry at 260 nm (BioPhotometer, Eppendorf, Le Pecq, France) before Reverse Transcription (RT). A 1 µg quantity of total RNAs was converted to cDNAs using an iScript cDNA synthesis kit (Bio-Rad, Marne-la-Coquette, France).

For qPCR, 4 µL of 100 × diluted cDNAs were added to 11 µL of 1 x SYBR Green Master Mix (Bio-Rad, Marne-la-Coquette, France) containing 0.5 µM of specific primers. Amplification reactions were performed with a real-time thermocycler (CFX96 Real Time System, Bio-Rad, Marne-la-Coquette, France) using the following a three-step program: an activation step at 95 °C for 3 min, 40 cycles of denaturation at 95 °C for 10 s, and finally, an extending step at 60 °C for 40 s. For each pair of primers, threshold values and PCR efficiency (≈100%) were determined using a range of serial cDNA dilutions. The single peak in the melting curves and the sequencing of the amplicon (Eurofins, Nantes, France) validated the specificity of the amplification for each primer pair. Gene expression in the leaves of plants exposed to a water deficit was calculated relative to the control with the ΔΔCt method using the following equation:

Relative expression = 2^−ΔΔCt^

with

ΔΔCt = ΔCt_sample_ − ΔCt_control_ (1)

and

ΔCt = Ct_target gene_ − Ct_housekeeping gene_ (2)

Using this method, the relative expression in the leaves of the target gene in the control sample was equal to 1 [104].

### 4.3. Transcriptomic Analysis by RNA-Sequencing (RNA-Seq)

As previously described [53], the RNA-seq samples were obtained from three independent replicates of total RNA extracted as described above with an Illumina NexSeq500 at the POPS platform of the Institute of Plant Science (IPS2) in Paris-Saclay (France). RNA-seq libraries were constructed using the TruSeq Stranded mRNA library prep kit (Illumina^®^, San Diego, CA, USA) according to the supplier’s instructions. The libraries were sequenced in paired-end (PE) mode with 75 bases for each read on an Illumina NextSeq500 to generate approximately 19 million PE reads per sample. Adapter sequences and bases with a Q-Score below 20 were trimmed out from reads using Trimmomatic (version 0.36, Illumina^®^, San Diego, CA, USA [105]) and reads shorter than 30 bases after trimming were discarded. Reads corresponding to rRNA sequences were removed using sortMeRNA (version 2.1, CRISTAL, Lille, France) [106]) against the silva-bac-16s-id90, silva-bac-23s-id98, silva-euk-18s-id95, and silva-euk-28s-id98 databases. Filtered reads were then mapped and counted using STAR (version 2.7.3a, [107]) with the following parameters --alignIntronMin 5 --alignIntronMax 60000 --outSAMprimaryFlag AllBestScore --outFilterMultimapScoreRange 0 --outFilterMultimapNmax 20-- alignEndsType Local on the *Brassica napus* genome (annotation V5 from Genoscope accessed on 2nd February 2021: http://www.genoscope.cns.fr/brassicanapus/data/) and its associated GTF annotation file. Between 90% and 93% of the reads were associated with annotated genes. Next, genes with less than 0.6 read per million in at least 3 of the samples were discarded, using the filterByExp function of the edgeR package. The resulting raw count matrix was fed into edgeR [108] for differential expression testing by fitting a negative binomial generalized log-linear model (GLM), including a replicate factor, a treatment factor, a time factor, and an interaction between treatment and time to the TMM-normalized read counts for each gene. Gene expression was compared between control and plants subjected to drought. The distribution of the resulting *p*-values followed the quality criterion previously described [109]. Genes with an adjusted *p*-value (FDR, Benjamini-Hochberg adjustment [110]) below 0.05 were considered as differentially expressed.

Genes were considered to be differentially expressed (DEG) for an adjusted *p*-value ≤ 0.05, whatever the absolute value of the “Log2 fold change” (Supplementary Data S2 and S3). Fragments Per Kilobase Million (FPKMs) were calculated for visual analysis only and were obtained by dividing normalized counts by gene length. PCA was performed with the mixOmics package (mixomics.org) (version 6.14.0), within R (R-project.org) (version 4.0.3) using log2-transformed normalized expression data. RNA-seq expression data were validated by using four DEGs with contrasting fold changes for RT-qPCR analysis. RT and qPCR were performed following the protocol described previously. Results of RT-qPCRs are presented in Supplementary Figure S4.

### 4.4. RNA-Seq Bioinformatic Analysis

Gene ontology enrichment analysis from Differentially Expressed Genes (DEGs) (Supplementary Data S3) was performed using the free web server of g:Profiler (biit.cs.ut.ee/gprofiler) [111] (version e104_eg51_p15_3922dba) with the Benjamin et Hochberg FDR correction method (threshold = 0.05). Then GO terms and associated adjusted *p*-values obtained from g:Profiler was inputted into the REVIGO tool in order to summarize the lists of GO terms [112] (version 01 February 2021, the setting was medium allowed similarity) (Supplementary Data S1).

Transporter-associated genes were selected from the *Arabidopsis thaliana* curated list of KIG [66] using the BioMart tool of the Ensembl Plants database (from Ensembl Plants Genes 51—*Arabidopsis thaliana* genes (Tair10)—*Brassica napus* genes (AT_PRJEB5043_v1) in order to find *Brassica napus* orthologs, and these were supplemented with a set of DEGs belonging to the GO term “ion transport” (GO:0006811) extracted from Amigo2 and unambiguously related to the transport of elements (Supplementary Data S2). 

Genes associated with biosynthesis and degradation of ABA, SA, GSH, GSSG, DHA, and proline were identified using the BIN hierarchical functional categories of the MapMan software (version 3.6.0RC1) [113,114], with genes mapped on *Brassica napus* annotation (version X4.2 brassica_napus from Ensembl Plants Genes 44) placed on summarized pathways according to the Plant Metabolic Network databases [115].

### 4.5. Untargeted Metabolic Profiling Using UPLC-MS/MS

The method used has been previously described [53]. Briefly, ground frozen leaf tissues (50 mg) were used for extraction, and the 1mL buffer for metabolite extraction contains 70% MeOH (Optima LCMS grade, Thermo Fisher Scientific, Waltham, MA, USA), 29% H_2_O (Milli-Q, 18.2 MΩ·cm, Millipore, MA, USA) and 1% formic acid (LCMS grade, Honeywell Fluka, Seelze, Germany). Samples were then centrifuged, and the supernatant was used for analysis by UPLC-MS/MS (Ultra performance liquid chromatography-Tandem mass spectrometry). Separation and detection were performed using an Acquity UPLC system (Waters, Milford, MA, USA) linked to a Xevo G2-S QTof mass spectrometer (Waters) equipped with a LockSpray electrospray ionization (ESI) source. Sample separation was accomplished by injecting 10 µL into an HSS T3 C18, 2.1 × 100 mm, 1.8 µm column (Waters), kept at 40 °C, at a flow rate of 0.5 mL min^−1^. The mobile phases were composed of two solvents (A: Milli-Q water containing 0.1% formic acid, and B: acetonitrile containing 0.1% formic acid). Optimal separation was obtained using the following gradient: 0–1 min at 98% A, 1–7 min from 98% to 0% A, maintained at 0% A to 9 min, 9–10 min from 0% to 98% A, maintained at 98% until 12 min for column regeneration. Mass spectrometry analysis was performed in positive and negative ionization modes using the following parameters: source voltage 0.5 kV (pos) and 2.5 kV (neg); cone voltage 40 V; source temperature 130 °C; desolvation gas temperature 550 °C; desolvation gas flow 900 L/h. Mass spectra were acquired in MS^E^ mode from 50 to 1200 *m*/*z* at 0.1 s scan^−1^. The ramp collision energy was set at 25 to 40 V. Samples were injected in randomized order. A quality control (QC) sample was prepared from an equal mix of all collected samples. The QC sample was injected every six samples to assess system stability.

After acquisition, metabolomic data were processed using Progenesis QI software (Version 3.0, Waters, Milford, MA, USA). Identification was carried out using the online open-source: Plant Metabolic Pathway Databases-Plant Metabolic Network (plantcyc.org) (Version 15.0.1) [116] with a mass tolerance of 10 ppm. For identified metabolites, the experimental MS^2^ spectrum was compared to the MS-DIAL reference MS/MS database, CompMS-MS-DIAL (riken.jp) (Version 14) [117] when possible; otherwise, it was compared to the theoretical fragmentation spectrum. The fragment mass tolerance is set at 10 ppm. Only identified metabolites were kept for further multivariate analysis.

Multivariate analysis was performed using EZInfo software (Version 3.0.3.0, Umetrics, Umeå, Sweden). Raw data were mean-centered, and Pareto scaled [118]. For each duration of water restriction, partial least squares discriminant analyses (PLS-DA) [119] were performed between water-deficient samples and their corresponding control samples. For each comparison, the influence of each metabolite on the classification was calculated by the variable influence on projection (VIP) [119,120]. Metabolites with VIP > 1 have an above-average influence. In this study, only metabolites with VIP > 2 were considered as being differentially expressed for further analyses. The metabolites list can be found in Supplementary Data S4.

For a better understanding of metabolic regulation induced by each duration of water restriction, pathway analysis was performed using the online open-source software, MetaboAnalyst 5.0 (metaboanalyst.ca) [121]. For each pathway, the *p*-values of metabolite set enrichment analysis and pathway impact of topology analysis were calculated using the KEGG database [122].

### 4.6. Phytohormone Analysis

Profiles of phytohormones were quantified on leaf samples harvested after 0, 5, 11, and 20 d of water restriction, with the samples being stored at −80 °C prior to analysis. Jasmonic acid (JA) and abscisic acid (ABA) standards were obtained from Sigma (Sigma-Aldrich, Saint-Louis, MI, USA), and the internal standard was labeled with stable isotopes (^2^H_5_-JA and ^2^H_6_-ABA) from OlchemIn (Olomouc, Czech Republic). Ground frozen leaves (20 mg) were extracted with 1 mL buffer containing 70% methanol (Optima LCMS grade, Thermo Fisher Scientific, Waltham, MA, USA), 1% formic acid (LCMS grade, Honeywell Fluka, Seelze, Germany), and 29% Milli-Q water containing internal standards labeled with stable isotope. The extracts were then centrifuged at 17,927× *g,* and the supernatants were collected. The supernatants were evaporated (SPE Dry 96, Biotage, Uppsala, Sweden) and resuspended in 2% formic acid solution and purified using an SPE ABN express column of 1 mL/30 mg (Biotage, Uppsala, Sweden). Phytohormones were then eluted using methanol, and samples were evaporated. Before injection into the UPLC-MS/MS system, the extract was resuspended in a 0.1% formic acid solution. Separation and detection were performed using a Nexera X2 UHPLC system (Shimadzu, Kyoto, Japan) linked to a QTrap 6500+ mass spectrometer (SCIEX, Concord, ON, Canada) equipped with an IonDrive turbo V electrospray source. Two µL were injected into a Kinetex Evo C18 core-shell column kept in an oven at 40 °C (100 × 2.1 mm, 2.6 µm, Phenomenex, Torrance, CA, USA) at a flow rate of 0.7 mL min^−1^ in order to separate phytohormones. The mobile phases were composed of 2 solvents (A: Milli-Q water containing 0.1% formic acid and B: acetonitrile LCMS grade containing 0.1% formic acid). Separation was done using the following gradient: a linear gradient from 1 to 60% B over 5 min; 60 to 100% B from 5 to 5.5 min; maintained at 100% B from 5.5 to 7 min; 100 to 1% B from 7 to 7.5 min; and maintained at 1% until 9.6 min for column regeneration. Analysis by mass spectrometry was achieved in negative mode and scheduled multiple reaction monitoring modes (MRM). Mass spectrometry acquisition was performed using the following parameters: ion spray voltage −4500 V; source temperature 600 °C; curtain gas 35 psi; nebulizer gas 50 psi; heater gas 60 psi; collision gas medium; entrance potential -10 V; MRM detection window 30 s; target scan time 0.075 s. The targeted MRM transitions are 209.0/59.0 (JA), 214.1/62.2 (D-JA), 263.0/153.0 (ABA), and 269.1/159.0 (D-ABA).

### 4.7. Proline Quantitative Analysis

The proline quantification method was adapted from Troll and Lindsley 1955 [123]. Fifty mg of ground frozen leaf tissues were extracted using 1 mL of ethanol (VWR, Rosny-sous-Bois, France). After extraction, samples were dried and resolubilized with 250 µL of water. After centrifugation, 100 µL of supernatant was added to 1 mL of 0.1 g/L ninhydrin solution in 60% acetic acid (Sigma-Aldrich, Saint-Louis, MI, USA). The mix was heated at 95 °C for 20 min, and 3 mL of toluene (Thermo Fisher Scientific, Waltham, MA, USA) was added to the mix after cooling. The organic (toluene) phase containing the red chromophore formed by ninhydrin and proline was kept for spectrophotometric measurement at 520 nm (SpectraMax i3x, Molecular Devices, San Jose, CA, USA).

### 4.8. Element Content Analysis by Mass Spectrometry and X-ray Fluorescence

Dried leaf samples were ground using 4 mm diameter inox beads in an oscillating grinder (Mixer Mill MM400, Retsch, Haan, Germany). The concentrations of most elements (Mg, P, S, K, Ca, B, Mn, Fe, Ni, Cu, Zn, Mo Na, Co, V, and Se) were quantified using the procedure previously described [102] using 40 mg of dry powder per sample that was submitted to acid digestion and mineralization. The resulting solutions were then analyzed by high-resolution inductively coupled plasma mass spectrometry (HR-ICP-MS, Element 2^TM^, Thermo Fisher Scientific, Bremen, Germany).

The total N concentration was determined with 1.5 mg of fine powder placed in tin capsules before analysis with an isotope-ratio mass spectrometer (IRMS, Isoprime, GV Instruments, Manchester, UK) linked to a C/N/S analyzer (EA3000, Euro Vector, Milan, Italy).

The remaining elements (Cl, Si, and Al) were quantified with approximately 1 g of dry weight powder analyzed with an X-ray-fluorescence spectrometer (XEPOS, Ametek, Berwyn, PA, USA) using calibration curves obtained from international standards with known concentrations.

In this study, elemental content is expressed as the ratio WD/control plants following the calculation method described [61].

### 4.9. Statistical Analysis

The experiment was performed with five independent replicates, each consisting of a pool of two individual plants, except for transcriptomic data, for which three independent replicates were used. Thus, plant biomass is indicated as the mean ± S.E. (*n* = 5), while nutrient content was given as the ratio WD/control (*n* = 5).

Statistical analyses were performed using the free acess R software (R-project.org) (version 4.0.3: R Core Team, 2020) and its extension RStudio (rstudio.com) (version 1.3.1093: RStudio Team, 2020). Data were analyzed using analysis of variance (ANOVA), and mean values were compared using Tukey’s HSD post-hoc test.

## Data Availability

RNAseq data were submitted to the Gene Expression Omnibus (GEO) international repository: http://www.ncbi.nlm.nih.gov/geo accessed on 23 July 2021; GEO accession: GSE179022.

## Supplementary Materials

The following supporting information can be downloaded at: https://www.mdpi.com/article/10.3390/ijms23020781/s1.
