# Supplementary material for: Transcriptomic, Metabolomic and Ionomic Analyses Reveal Early Modulation of Leaf Mineral Content in Brassica napus under Mild or Severe Drought
Source: Int J Mol Sci. 2022 Jan 11;23(2):781. doi: 10.3390/ijms23020781 (PMC8776245; doi:10.3390/ijms23020781)
Supplement: Supplementary file 1 [file ijms-23-00781-s001.zip › Suplementary Table S1.pdf]

Supplementary Table S1: Physical and chemical soil properties

Soil properties	Methods of anaysis	Value
Exchangeable CaO (‰)	NFX 31-108 - ICP AES	2.14
Total nitrogen (‰)	NF ISO 13 878 - Dumas method	1.22
Exchangeable MgO (‰)	NFX 31-108 - ICP AES	0.46
P_2O_5 (‰)	NFX 31-160 - Dyer method	0.18
Exchangeable K_2O (‰)	NFX 31-108 - ICP AES	0.093
SO_3 (KH_2PO_4) (mg/Kg)	Extr. KH2PO4 - ICP AES	186
P_2O_5 (‰)	NFX 31-161 - Joret Hébert method	0.076
Exchangeable Na_2O (‰)	NFX 31-108 - ICP AES	0.035
P_2O_5 (‰)	NF ISO 11263 - Olsen method	0.048
Cu EDTA (mg/Kg)	NFX 31-120 - ICP AES	1.2
Soluble boron Eau (mg/Kg)	NFX 31-122	0.16
Fe EDTA (mg/Kg)	NFX 31-120 - ICP AES	49.1
Mn EDTA (mg/Kg)	NFX 31-120 - ICP AES	23.8
Zn EDTA (mg/Kg)	NFX 31-120 - ICP AES	1.4
Clay (‰)	NFX 31-107 - without decarbonatation	31
C/N	NF ISO 13 878 - Dumas method	42.9
Organic carbon (‰)	NF ISO 14 235	52.3
CEC (me/Kg)	NFX 31-130 - Metson method	110
Fine silt (s.déc) (‰)	NFX 31-107 - without decarbonatation	24
Coarse silt (s.déc) (‰)	NFX 31-107 - without decarbonatation	6.1
Organic matter (‰)	NF ISO 14 235	90.4
Water pH	NF ISO 10 390	6.2
KCl pH	NF ISO 10 390	5.9
Fine sand (‰)	NFX 31-107 - without decarbonatation	36
Coarse sand (‰)	NFX 31-107 - without decarbonatation	903
