# Supplementary material for: Transcriptomic, Metabolomic and Ionomic Analyses Reveal Early Modulation of Leaf Mineral Content in Brassica napus under Mild or Severe Drought
Source: Int J Mol Sci. 2022 Jan 11;23(2):781. doi: 10.3390/ijms23020781 (PMC8776245; doi:10.3390/ijms23020781)
Supplement: Supplementary file 1 [file ijms-23-00781-s001.zip › Suplementary Table S2.pdf]

Supplementary Table S2: Composition of the nutrient solution derived from Hoagland nutrient solution and adapted to ensure the plants' mineral needs during the experiment. Elemental concentrations were kept balanced in solution to provide 40 kg N ha-1 to ensure a non-limiting mineral condition for all the experiments.

Salts	Salt concentrations (µM)
KNO_3	1000
$Ca(NO_3)_2 + 4H_2O$	1250
$Si(OH)_4$	900
HCl	600
$CaCl_2 + 2H_2O$	500
$MgSO_4$	400
$CaCO_3$	360
KH_2PO_4	200
Na_2SiO_3	200
K_2SO_4	100
KCl	100
NaFe-EDTA $3H_2O$	50
NaFe-EDDHA	50
H_3BO_3	10
$MnSO_4$	3
$ZnSO_4$	3
$CuSO_4$	0.7
$NiCl_2$	0.15
$CoCl_2$	0.1
Na_2SeO_4	0.01
$(NH_4)_6Mo_7O_{24}$	0.008

Nutrients	Elemental concentrations in solution (µM)	input kg ha^{-1}
N	3500	40
Mg	400	8
P	200	5
S	507	13
K	1500	48
Ca	2110	69
B	10	0.09
Cl	2001	58
Mn	3	0.13
Fe	100	5
Ni	0.15	0.007
Cu	0.7	0.04
Zn	3	0.16
Mo	0.056	0.004
Na	500	9
Al		
Si	1100	25
V		
Co	0.1	0.005
Se	0.01	0.001
