# Supplementary material for: Transcriptomic, Metabolomic and Ionomic Analyses Reveal Early Modulation of Leaf Mineral Content in Brassica napus under Mild or Severe Drought
Source: Int J Mol Sci. 2022 Jan 11;23(2):781. doi: 10.3390/ijms23020781 (PMC8776245; doi:10.3390/ijms23020781)

Supplementary Figure S4: (A) Expression profiles of target genes used for RNAseq validation; (B) Spearman correlation and confidence interval between log2 fold change ratio from RNA-seq and RT-qPCR.

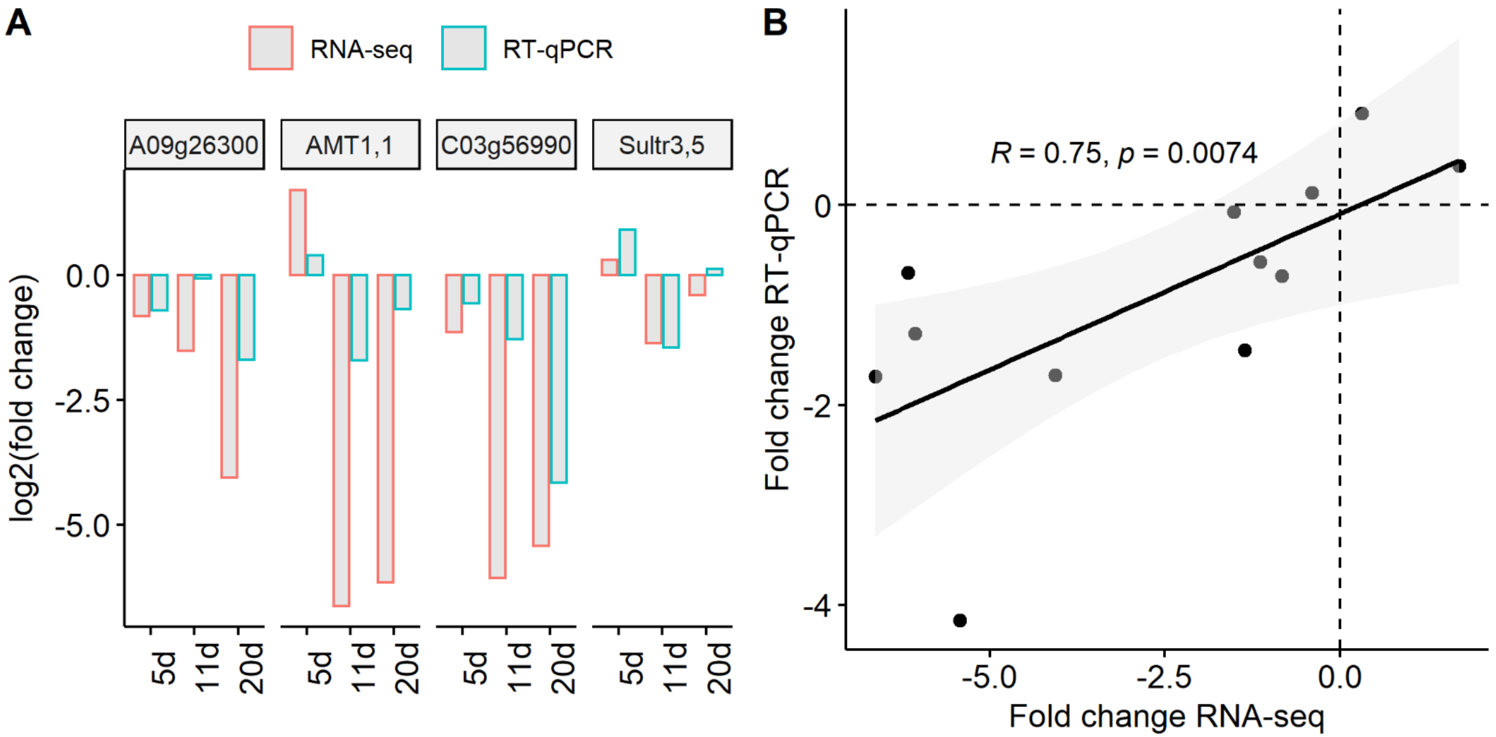

Supplement: Supplementary file 1 [file ijms-23-00781-s001.zip › Supplementary Figure S4.pdf]
