# Supplementary figures and images for: Transcriptomic, Metabolomic and Ionomic Analyses Reveal Early Modulation of Leaf Mineral Content in Brassica napus under Mild or Severe Drought
Source: Int J Mol Sci. 2022 Jan 11;23(2):781. doi: 10.3390/ijms23020781 (PMC8776245; doi:10.3390/ijms23020781)

REVIGO TreeMap

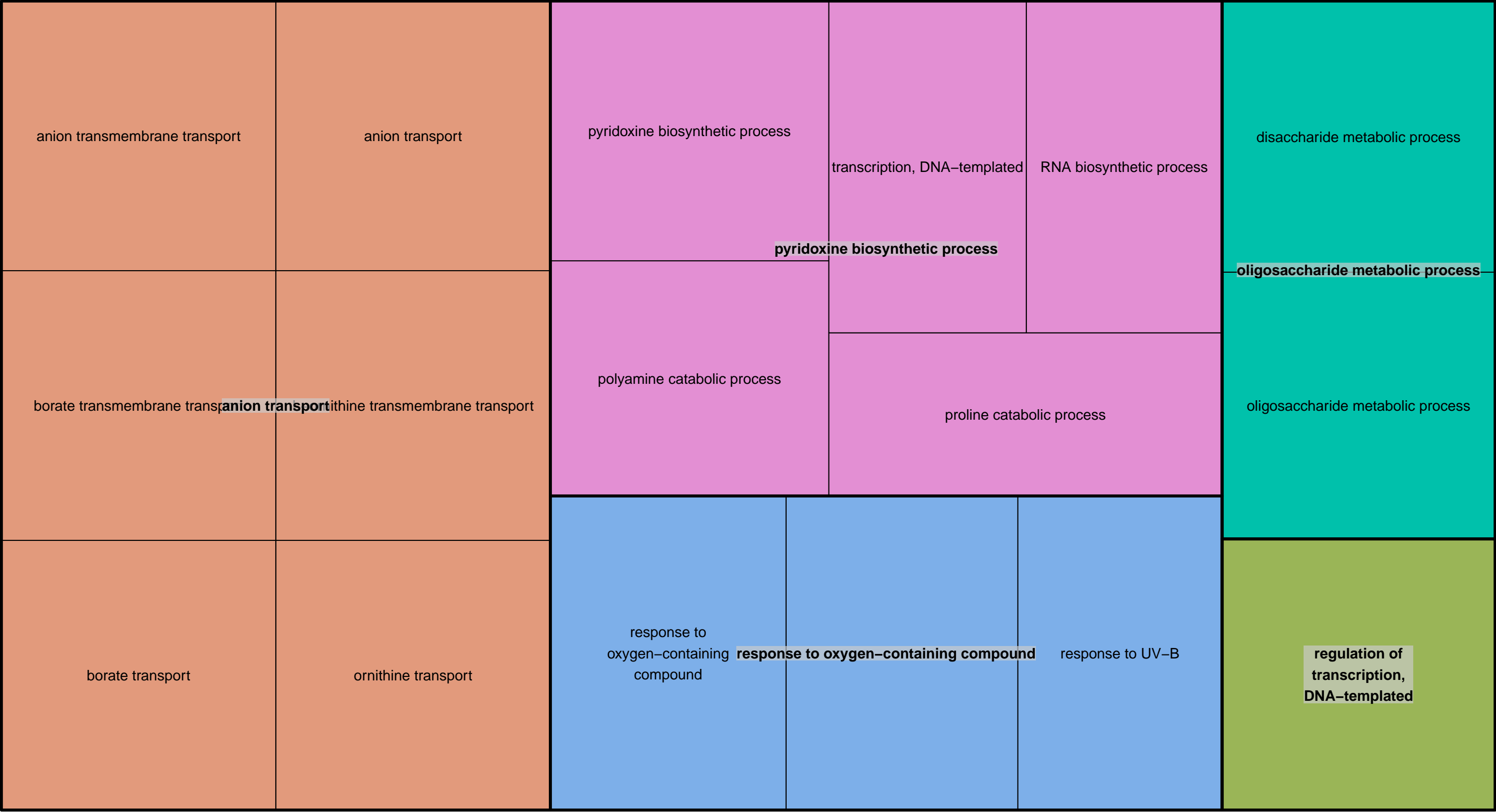

Supplement: Supplementary file 1 [file ijms-23-00781-s001.zip › Supplementary Figure S1.pdf]

REVIGO TreeMap

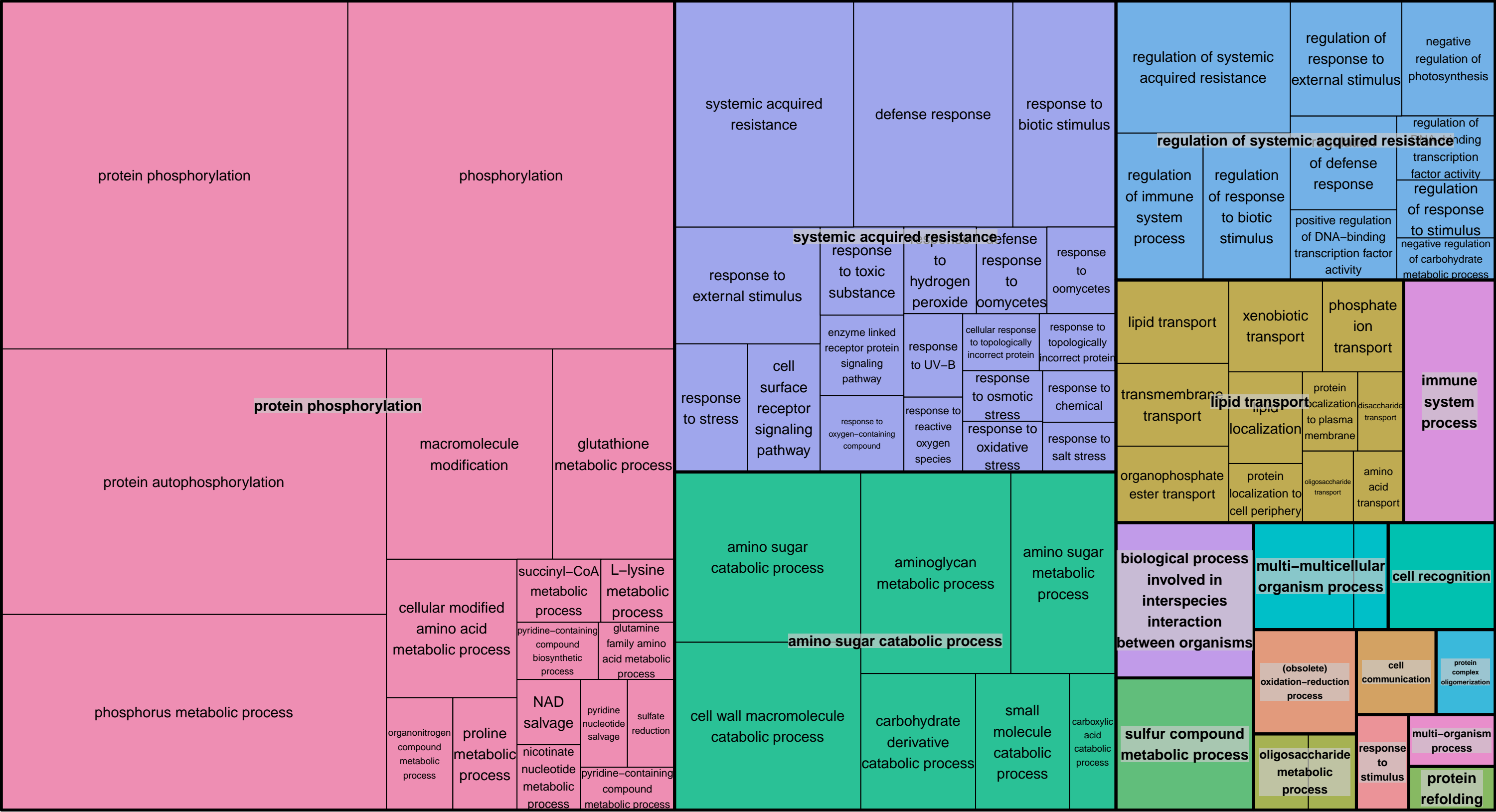

Supplement: Supplementary file 1 [file ijms-23-00781-s001.zip › Supplementary Figure S2.pdf]

REVIGO TreeMap

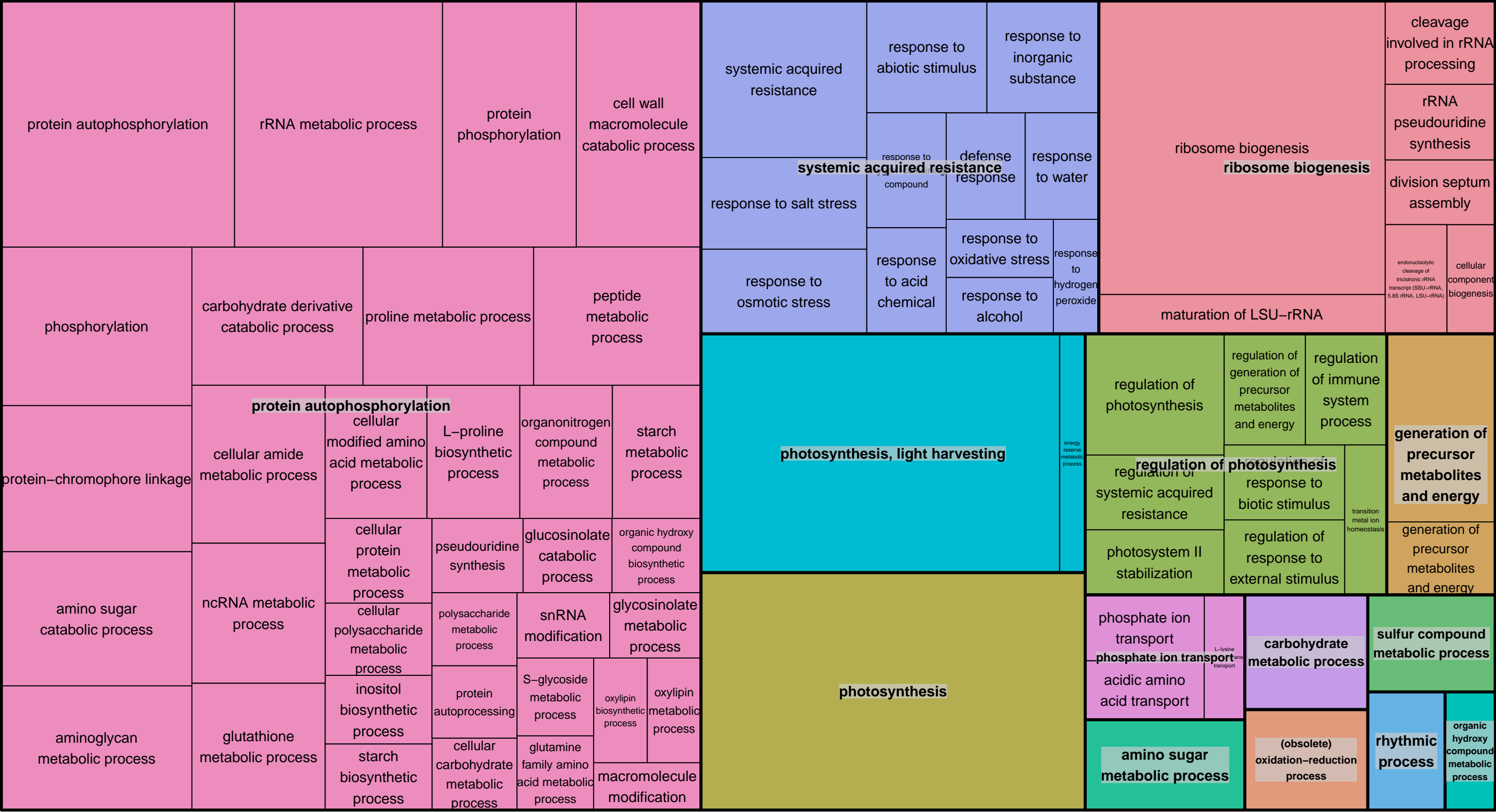

Supplement: Supplementary file 1 [file ijms-23-00781-s001.zip › Supplementary Figure S3.pdf]
